# Convenient Preparation of ^18^F-Labeled Peptide Probes for Potential Claudin-4 PET Imaging

**DOI:** 10.3390/ph10040099

**Published:** 2017-12-18

**Authors:** Lucia Feni, M. Aymen Omrane, Moritz Fischer, Boris D. Zlatopolskiy, Bernd Neumaier, Ines Neundorf

**Affiliations:** 1 Department of Chemistry, Biochemistry, Zülpicher Str. 47a, 50674 Cologne, Germany; lfeni@uni-koeln.de (L.F.); mfisch11@smail.uni-koeln.de (M.F.); 2 Institute of Neuroscience and Medicine, INM-5: Nuclear Chemistry, Forschungszentrum Jülich GmbH, Wilhelm-Johnen Str., 52425 Jülich, Germany; a.omrane@fz-juelich.de (M.A.O.); boris.zlatopolskiy@uk-koeln.de (B.D.Z.); 3 Institute of Radiochemistry and Experimental Molecular Imaging, University Clinic Cologne, Kerpener Str. 62, 50937 Cologne, Germany; 4 Max Planck Institute for Metabolism Research, Gleueler Str. 50, 50931 Cologne, Germany

**Keywords:** pancreatic cancer, PET-imaging, claudin receptors, targeting peptides, 5-[^18^F]FDR, ^18^F-labeling

## Abstract

Since pancreatic cancer is often diagnosed in a late state of cancer development, diagnostic opportunities allowing early disease detection are highly sought after. As such, cancer expression of claudin proteins is markedly dysregulated, making it an attractive target for molecular imaging like positron emission tomography (PET). Claudins are a family of transmembrane proteins that have a pivotal role as members of the tight junctions. In particular, claudin-3 and claudin-4 are frequently overexpressed in pancreatic cancer. ^18^F-Labeled claudin selective peptides would provide access to a novel kind of imaging tools for pancreatic cancer. In this work we describe the synthesis of the first ^18^F-labeled probes potentially suitable for PET imaging of claudin-4 expression. These probes were prepared using oxime ligation of 5-[^18^F]fluoro-5-deoxyribose (5-[^18^F]FDR) to claudin selective peptides. As a proof-of-principle, one of them, 5-[^18^F]FDR-Clone 27, was isolated in >98% radiochemical purity and in 15% radiochemical yield (EOB) within 98 min, and with a molar activity of 4.0 GBq/μmol (for 30 MBq of tracer). Moreover, we present first biological data for the prepared 5-FDR-conjugates. These tracers could pave the way for an early diagnosis of pancreatic tumor, and thus improve the outcome of anticancer therapy.

## 1. Introduction

Since pancreatic cancer rarely causes symptoms in early stages, its treatment is often challenging. Usually, if discovered, the tumor is already in a metastatic state and seldom resectable. Only 15–20% of the tumors can be completely removed so that the five-year survival rate for patients with pancreatic cancer is only about 8%. The average life expectancy after detecting tumor metastasis is less than half a year [1,2]. Thus, early diagnosis of pancreatic cancer is highly important, and different imaging techniques are applied [3]. Besides computed tomography (CT), endoscopic ultrasound (EUS) and magnetic resonance imaging (MRI), positron emission tomography (PET) is also applied. PET is a widely used non-invasive molecular imaging modality in clinical diagnostics. It enables the visualization of physiological and pathological processes in vivo with high resolution by means of probes labeled with an appropriate β^+^-emitting radioisotope. Owing to its favorable decay properties, fluorine-18 is the most commonly used PET-radionuclide, which includes very low positron energy (630 keV) and a half-life (109 min) appropriate for the majority of PET applications. Generally, PET imaging is particularly well suited to detect tumors at an early stage, as well as metastasis [3]. The work horse in clinical PET imaging is still 2-[^18^F]fluoro-2-deoxy-d-glucose (2-[^18^F]FDG). Unfortunately, the reported sensitivity and specificity of FDG-PET for the delineation of pancreatic cancer are only 46–71% and ≥63%, respectively [4]. To increase the sensitivity and selectivity of PET imaging and to realize an efficient diagnostic method, research focused on the development of peptide-based PET tracers in recent years. Peptides comprise specific targeting properties, are often non-toxic, and can be easily synthesized and chemically modified [5]. 

**Figure 1:**
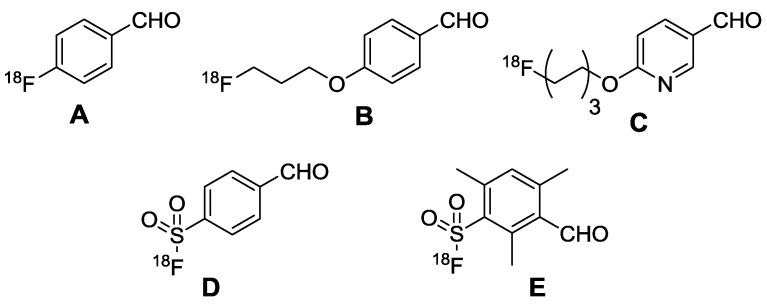
^18^F-Labeled aldehydes for peptide conjugation. (**A**) 4-[^18^F]fluorobenzaldehyde (4-[^18^F]FBA); (**B**) 4-{3-([^18^F]fluoro)propoxy}benzaldehyde; (**C**) 6-{2-([^18^F]fluoro)ethoxy}nicotinaldehyde; (**D**) 4-formylbenzenesulfonyl [^18^F]fluoride; (E) 3-formyl-2,4,6-trimethylbenzenesulfonyl [^18^F]fluoride.

The efficient conjugation of an appropriately radiofluorinated building block to the peptide can be realized by using various bioconjugation techniques [6,7]. Among them, the chemoselective oxime ligation that enables conjugation of ^18^F-labeled aldehydes (some examples are depicted in Figure 1) to aminooxy-functionalized peptides has gained great popularity [8,9].

4-[^18^F]Fluorobenzaldehyde (Figure 1, compound **A**) is the most frequently used prosthetic group for peptide labeling. Furthermore, many other aromatic aldehyde functionalized prosthetic groups have been described in the literature. However, the presence of the hydrophobic aromatic moieties may cause an increase of the overall lipophilicity of the radiolabeled biomolecules, and, consequently, adversely affect their pharmacokinetic properties [10,11]. On the other side, aldose sugars (e.g., glucose, ribose) represent an interesting hydrophilic building block suitable for peptide conjugation. Recently, it has been shown that under appropriate reaction conditions 5-[^18^F]fluoro-5-deoxy-d-ribose (5-[^18^F]FDR) can be conjugated to biopolymers much more efficiently and rapidly than other aldoses like 2-FDG [11]. 

Within this study, 5-[^18^F]FDR was used to prepare ^18^F-labeled peptide conjugates with high affinity to claudin-4, a member of the claudin family, which is highly overexpressed in pancreatic tumor tissues. The claudins are transmembrane proteins and important members of the ‘tight-junction’ (TJ), which regulates the permeability of paracellular barriers [12]. Depending on the cell type, the TJ is differently permeable to solutes and limits diffusion of membrane lipids and proteins through the cell membrane affecting the polarity of cells [13]. Until now, 27 different claudins, having all a similar structure, were identified [14]. They consist of four transmembrane domains, two extracellular and intracellular loops, with the *C*- and *N*-terminal domains located in the cytoplasm [15]. The largest extracellular loop (ECL-1) can interact with the ECL-1 of an adjacent cell and thus build a portion of the TJ.

Claudins are promising targets for cancer imaging and therapy, as they are differently expressed in tumors compared to normal tissues. In most tumors (e.g., claudin-1 in breast cancer cells) their expression is reduced, leading to diminished adhesion and deformation of tumor cells [16]. In contrast, in pancreatic cancer cells claudin-4 is overexpressed leading to increased permeability, higher osmotic stress and greater permeation of solutes [17,18]. The overexpression of claudin-4 begins at a very early stage of cancer progression, precisely within pancreatic intraepithelial neoplasia, the precursor lesion of pancreatic cancer, and thus offers an excellent opportunity for early detection [18,19,20,21,22,23]. Recently it has been found that the second loop (ECL-2) of some claudins (such as claudin-4) is a receptor for *Clostridium perfringens* enterotoxin (CPE). CPE is one of the 13 toxins that are produced by the anaerobic bacterium *C. perfringens*. It is a 35 kDa polypeptide, which is highly toxic to humans at very low concentrations [24]. Due to the affinity to claudins, CPE could be a potential tool to target pancreatic cancer. However, since CPE is too toxic for many cell studies, Sonoda et al. developed a non-toxic variant (C-CPE) that is a *C*-terminal fragment of CPE (184–319) still bearing high affinity to claudin-4 (K_a_ 1.0 × 10^8^ mol·L^−1^) [25,26]. On the basis of this sequence, several research groups developed peptides with selectivity towards various claudins. Moreover, by using “phage display” techniques, Ling et al. identified peptides, including CC4P-5, which possess even higher affinity to claudin-4 than C-CPE and analogs thereof [27,28].

The aim of this work was the preparation and preliminary biological evaluation of peptide conjugates potentially suitable for PET-imaging of infiltrating pancreatic ductal adenocarcinoma (PDAC), using claudin-4 as a molecular target. To the best of our knowledge, no reliable and efficient radiolabeling method for claudin-4 selective peptide based PET-tracers has been described in literature until now. Recent attempts relied on the use of a [^111^In]anti-claudin-4 mAb for diagnosis of pancreatic ductal carcinomas [29], or a [^64^Cu]-labeled CPE fragment that has been used for ovarian cancer diagnosis [30]. We introduced the ^18^F label via oxime ligation between 5-[^18^F]FDR and aminooxy-functionalized peptides. This radiolabeling strategy described by Li et al. [11] was optimized by us within the current study. We also studied the secondary structure of 5-FDR peptide conjugates in solution and determined their cellular toxicity.

## 2. Results and Discussion

### 2.1. Synthesis and Characterization of 5-FDR-Peptides

**Figure 2:**
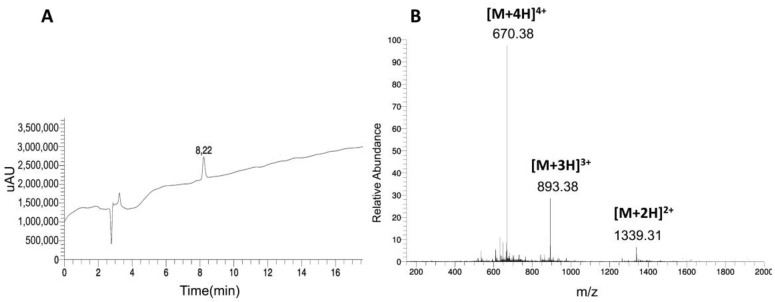
Analytical data of 5-FDR-C-CPE-17-KKK (**1**): (**A**) HPLC chromatogram obtained with a gradient of 10% to 60% acetonitrile-water over 20 min; (**B**) ESI-MS spectrum of the compound (see experimental section for more details).

In recent studies, several claudin-4 selective peptide binders were identified [25,26,27,28]. We used the reported sequences directly for the establishment of a reliable ^18^F-labeling strategy. Only peptide **1**, namely C-CPE-17, was slightly modified by introducing three lysines at the *C*-terminal to increase its overall solubility. All peptides were synthesized by standard Fmoc/*t*Bu solid phase peptide synthesis protocols. At the *N*-terminus we introduced aminooxyacetic acid (AOA) connected with the main peptide sequence via β-alanine as a short spacer. In a first set of experiments, all peptides were coupled with unlabeled 5-FDR to analyze the influence of the sugar residue on secondary structure, as well as cytotoxicity of the conjugates to two different cell lines. The identity and purity of the novel 5-FDR peptide conjugates was confirmed by LC-ESI MS (Table 1 and Figure 2).

**Figure 3:**
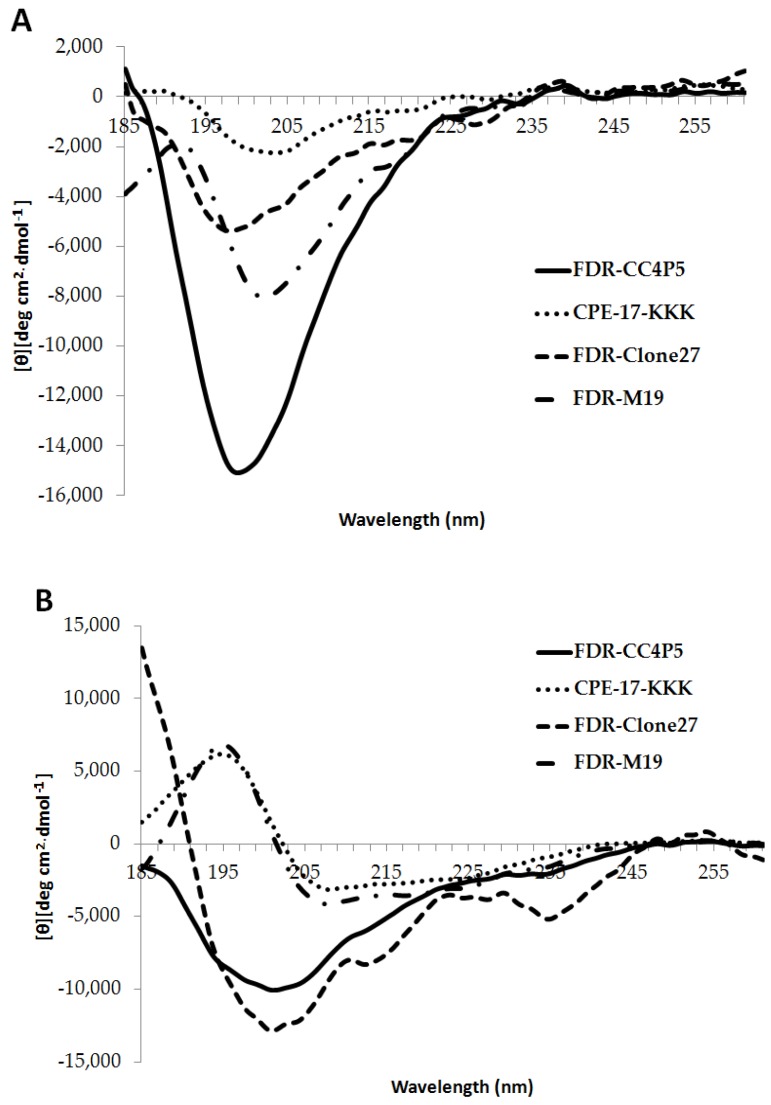
Circular dichroism spectra of the investigated peptides. The spectra were measured at a peptide concentration of 20 μmol·L^−1^ in 10 mmol·L^−1^ phosphate buffer (**A**) and 10 mmol·L^−1^ phosphate buffer with the additon of 50% TFE (**B**).

To get more insights into the secondary structure of the peptides in solution, CD spectroscopy analysis was performed. In phosphate buffer we recognized the typical spectrum of an unordered conformation for all peptides, while the addition of trifluoroethanol (TFE) supported the formation of a secondary structure. In fact, only the spectra of 5-FDR-C-CPE-17-KKK and 5-FDR-M19 showed values characteristic for the formation of an alpha helix (Figure 3). However, the shorter conjugates, 5-FDR-CC4P-5 and 5-FDR-Clone 27, were still present in random coil structure. This observation can be explained by the short length of their amino acid sequences.

### 2.2. Preliminary Biological Evaluation

**Figure 4:**
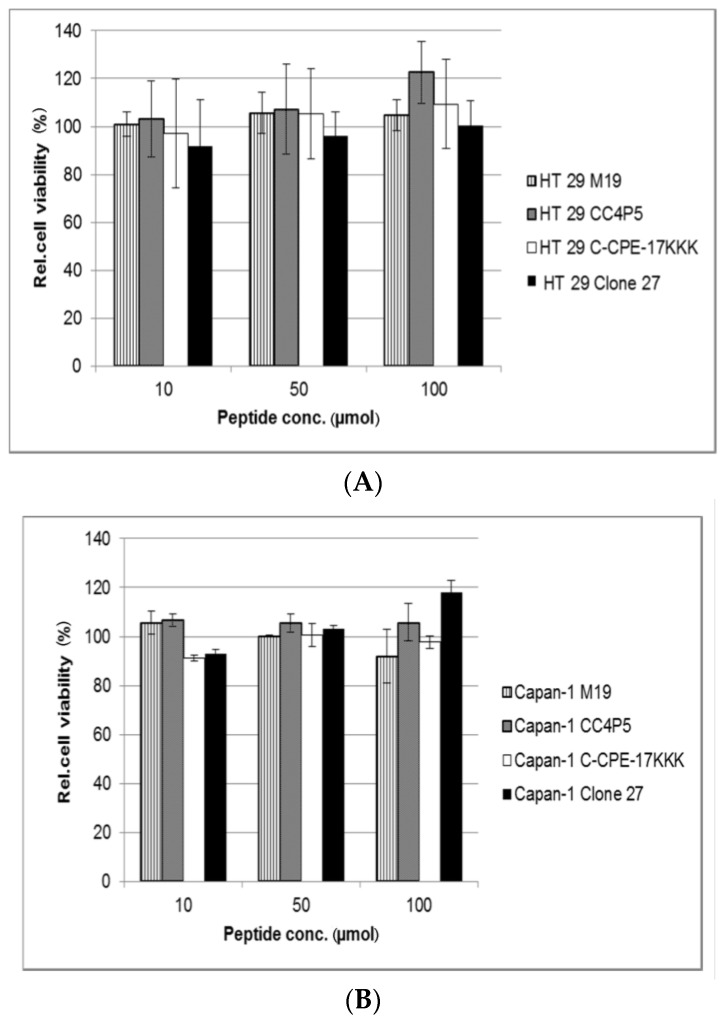
Cytotoxicity assay of HT-29 (**A**) and Capan-1 (**B**) cell lines. Solutions of 5-FDR-peptide conjugates (10, 50, 100 µmol·L^−1^) were incubated with the respective cells at 37 °C for 24 h.

Next, we studied the cytoxicity of the novel 5-FDR coupled peptides in Capan-1 and HT-29 human adenocarcinoma epithelial cells. Both cell lines are reported to overexpress claudin-4 endogenously, albeit to different extents [31]. Toxicity studies were performed by incubating the cells with the 5-FDR peptide conjugates for 24 h. No significant toxic effects were observed within the studied concentration range (10–100 µmol·L^−1^) (Figure 4). Therefore, all conjugates were suitable for further biological studies.

### 2.3. Optimization of the Preparation of 5-[^18^F]FDR Peptide Conjugates

**Scheme 1:**
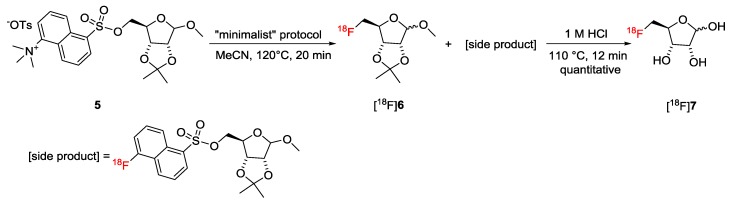
Preparation of 5-[^18^F]FDR under ‘minimalist’ conditions.

Next, we prepared 5-[^18^F]FDR using the recently developed ‘minimalist’ protocol for radiofluorination (Scheme 1) [10,32]. The term ‘minimalist’ was coined due to the exceptional simplicity of this protocol. Thus, according to the conventional procedure of nucleophilic radiofluorination [^18^F]fluoride produced in [^18^O]H_2_O is trapped on an anion-exchange resin to remove the bulk of water and then eluted with an aqueous solution of a base, such as K_2_CO_3_. Then, phase transfer catalyst such as 2.2.2-cryptand in acetonitrile is usually added and water is removed by tedious and time-consuming (typically, 15–20 min) azeotropic drying. Finally, to obtain the ^18^F-labeled target compound, the dried residue of [^18^F]KF/K_2_CO_3_/K2.2.2 complex is taken up in a solution of the precursor in a polar aprotic solvent and heated. In contrast, following the ‘minimalist’ method, ^18^F^−^ is eluted directly with a solution of the corresponding onium salt precursor in MeOH. Low boiling MeOH is completely removed within 3–5 min. Base and other additives are also not required. Consequently, the radiolabeling step is carried out using only the suitable onium salt precursor and [^18^F]fluoride.

**Figure 5:**
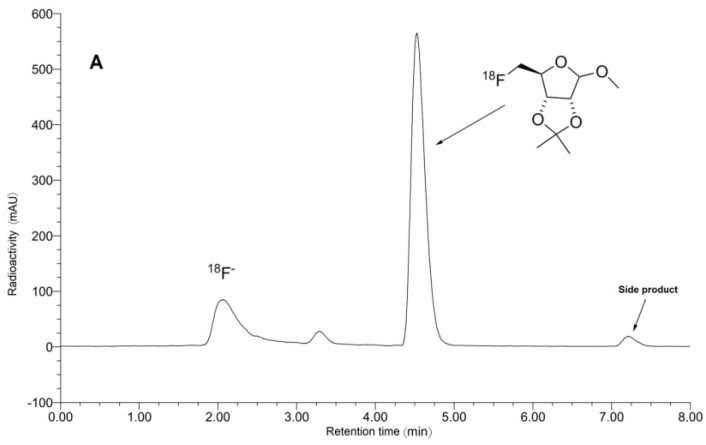

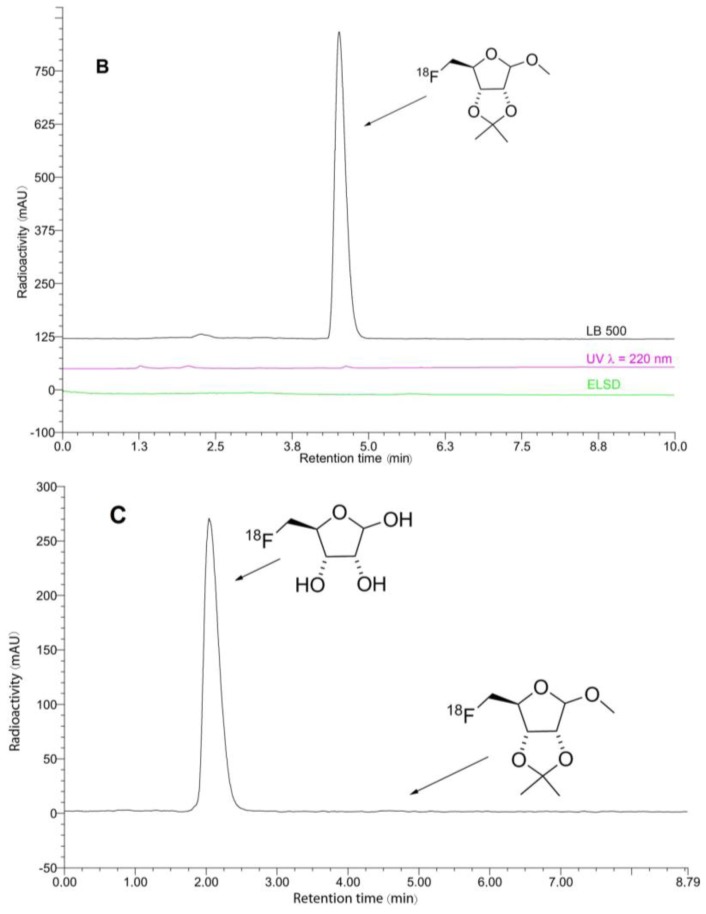
HPLC chromatograms of the crude and purified [^18^F]**6** ((**A**,**B**), respectively), and 5-[^18^F]FDR ([^18^F]**7**, (**C**)). Conditions: column: Phenomenex Luna C18(2), 250 × 4.6 mm, 5 µm; eluent: 0–5.5 min 55 → 95% MeCN; 5.5–8.0 min 95% MeCN; flow rate: 1.3 mL/min; *t*r = 4.5 min ([^18^F]**6**), *t*r = 2.0 min (5-[^18^F]FDR). Radioactivity, UV- (λ = 210 nm), and ELSD-traces are shown in black, violet, and green, respectively.

Accordingly, a solution of ^18^F^−^ in [^18^O]H_2_O was loaded onto an anion exchange resin and the resin was washed with MeOH, dried with air, and [^18^F]fluoride was eluted using a solution of tosylate **5** in MeOH (recovery of ^18^F^−^ >92%). After removal of MeOH the residue was taken up in acetonitrile and the solution was heated at 120 °C for 20 min affording the protected 5-[^18^F]FDR ([^18^F]**6**) in radiochemical conversions (RCCs) [33] of up to 60%, along with the formation of 3–5% side product presumably owing to the concurrent SNAr [^18^F]fluorination of 5-*N*,*N*,*N*-trimethylaminonaphthyl-1-sulfonyl residue (Figure 5). Noteworthy, with the corresponding triflate precursor RCCs did not exceed 35%. d-Ribose formed from the unreacted radiolabeling precursor **5** and side product during the deprotection step would compete with 5-[^18^F]FDR in oxime formation. Accordingly, intermediate [^18^F]**6** was purified by HPLC. The consequent hydrolysis with 1 mol·L^−1^ HCl at 110 °C for 12 min furnished 5-[^18^F]FDR in excellent radiochemical purity (RCP) >99% and radiochemical yield (RCY) [33] of 30% in two steps.

**Figure 6:**
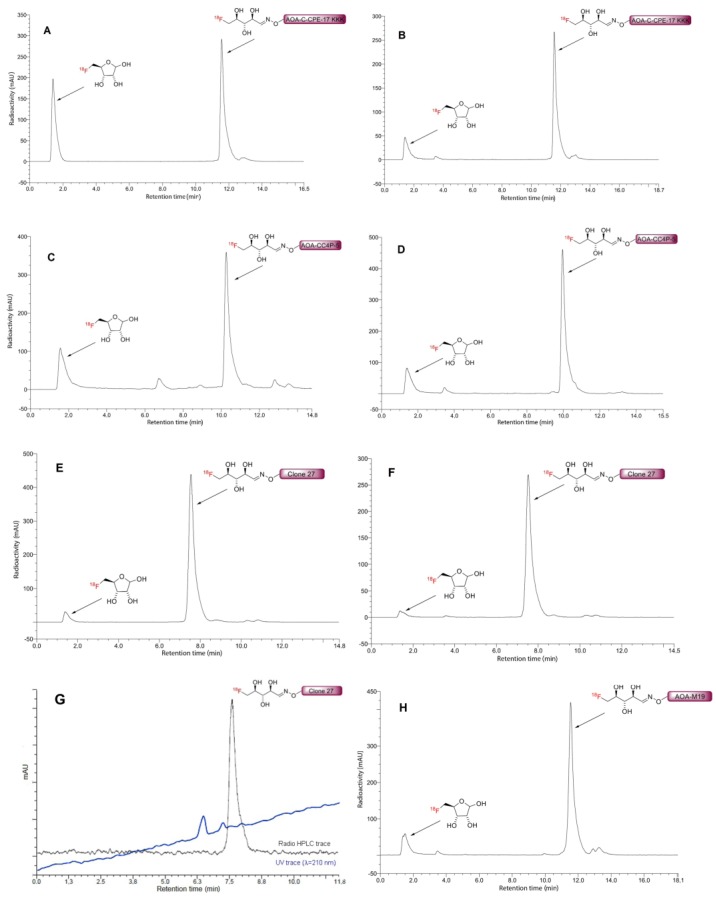
HPLC chromatograms of the crude 5-[^18^F]FDR-AOA-peptide conjugates prepared using ammonium acetate ((**A**,**C**,**E**); reaction conditions I) and anilinium acetate ((**B**,**D**,**F**,**H**); reaction conditions II) as well as purified 5-[^18^F]FDR-AOA-Clone 27 ([^18^F]**11**, (**G**)). Reaction conditions: I: 0.2 mol·L^−1^ NH_4_OAc buffer, pH 4, 75 °C, 20 min; II: 0.25 mol·L^−1^ anilinium acetate buffer, pH 4.6, RT, 10 min, for the applied amounts of AOA-peptides, refer to Table 2 and Table 3. HPLC conditions: column: Phenomenex Kinetex C18 100 × 4.6 mm, 2.6 μm/100 Å; eluent: 0–15 min 10 → 40% MeCN (0.1% TFA); flow rate: 1 mL/min; *t*r: [^18^F]FDR-AOA-C-CPE-17-KKK ([^18^F]**8**, (**A**,**B**))—11.5 min, [^18^F]FDR-AOA-CC4P-5 ([^18^F]**10**, (**C**,**D**))—10.3 min, [^18^F]FDR-AOA-Clone 27 ([^18^F]**11**, (**E**,**F**))—7.5 min, [^18^F]FDR-AOA-M19 ([^18^F]**9**, (**H**))—11.6 min, side product: 5-[^18^F]FDR—1.4 min. Radioactivity and UV- (λ = 210 nm; only (**G**)) traces are shown in black and violet, respectively.

Next, we examined the conjugation of 5-[^18^F]FDR with different aminooxy-functionalized claudine-4 binding peptides in ammonium acetate buffer (Table 2, Figure 6).

In this case, efficient conjugation was observed only at elevated temperature. With AOA-C-CPE-17KKK ([^18^F]**8**) and AOA-Clone 27 ([^18^F]**11**) the highest RCCs of 59% and 91%, respectively, were achieved using 1.0 µmol of the corresponding peptide. In contrast, reasonable RCCs of 5-[^18^F]FDR-AOA-M19 ([^18^F]**9**) and 5-[^18^F]FDR-AOA-CC4P-5 ([^18^F]**10**) conjugates (46% and 60%, respectively), were observed only if ≥1.6 µmol of the corresponding aminooxy-functionalized substrate was applied.

Recently, it has been shown that the oxime formation can be significantly accelerated by aniline catalysis [34,35,36]. Consequently, the oxime conjugation of 5-[^18^F]FDR and claudine-4 ligands in anilinium acetate buffer was studied (Table 3, Figure 6). 

Anilinium acetate buffer significantly increased the efficacy of oxime ligation. The desired radiolabeled conjugates were obtained in RCCs of 76–93% at ambient temperature within 10 min of reaction time. Extended reaction times and higher reaction temperature up to 70 °C did not affect the RCCs.

## 3. Materials and Methods

### 3.1. Materials

*N*^α^-Fmoc-protected amino acids, Oxyma Pure^®^, diisopropylcarbodiimide (DIC), trifluoroacetic acid, and 4-(2′,4′-dimethoxyphenyl-Fmoc-aminomethyl)phenoxy (Rink amide) resin were purchased from IRIS Biotech GmbH (Marktredwitz, Germany), Boc-aminooxyacetic acid, bis-Boc-aminooxyacetic acid, sodium hydroxide, potassium hydrogen phosphate, potassium dihydrogen phosphate, and HPLC pure water from Merck KgaA (Darmstadt, Germany), *tert*-butanol, acetic acid, sodium acetate, piperidine and triisopropylsilane (TIS) from Sigma-Aldrich (St. Louis, MO, USA). Diisopropylethylamine (DIPEA) and trifluoroethanol (TFE) were obtained from Alfa Aesar GmbH (Karlsruhe, Germany), 1,2-ethandithiole and thioanisole from Fluka Analytical/Sigma Aldrich (St. Louis, MO, USA), formic acid, ninhydrine, *N*-methyl-2-pyrrolidone (NMP), phenol from Carl Roth GmbH (Karlsruhe, Germany). Acetonitrile, dichloromethane, diethyl ether, dimethylformamide, ethanol, and methanol were from VWR BDH Prolabo (Radnor, PA, USA). The following side chain protecting groups were used: 2,2,4,6,7-pentamethyldihydrobenzofuran-5-sulfonyl (Pbf) for Arg; Trityl (Trt) for Asn, His and Gln; *tert*-Butyl (*t*Bu) for Asp, Ser, Tyr, Thr and Glu; *tert*-butyloxycarbonyl (Boc) for Lys and Trp.

The following cell media and supplements were used: Dulbecco’s modified Eagle’s medium (DMEM) with fetal bovine serum (FBS) and McCoy’s 5A Medium with FBS. Tissue culture 96-well plates used for resazurin cytotoxicity assay were obtained from Sarstedt Inc. (Newton, MA, USA), cell culture plates 100 × 20 mm were from BD Falcon (Franclin Lakes, NJ, USA).

### 3.2. Peptide Synthesis

All peptides were prepared by using an automated multiple solid-phase peptide synthesizer (Syro, MultiSynTech, Witten, Germany) according to the Fmoc-*t*Bu strategy. The peptides were synthesized as *C*-terminal amides using the Rink amide resin (0.48 mmol peptide per gram resin). β-Alanine and aminooxyacetic acid were coupled manually to the peptides using 3 eq. of the reagent, 3 eq. Oxyma and 3 eq. DIC for 2 h and the procedure was repeated twice. After the coupling with aminooxyacetic acid the peptide amides were finally cleaved from the resin using TFA:water:TIS (95:2.5:2.5, *v*/*v*/*v*) or TFA:TIS:EDT (90:3:7, *v*/*v*/*v*), for the peptide containing Trp (CC4P-5). 5 eq. of Boc-aminooxyacetic acid were added in the cleavage cocktail in order to avoid the formation of the acetone adduct with mass +40 [37]. After 3 h at room temperature, the peptides were precipitated in ice cold diethyl ether and then washed and centrifuged five times; the pellet was lyophilized from water:*tert*-butyl alcohol (3:1 *v*/*v*) and analyzed by RP-HPLC-ESI-MS on a Kinetex C18 column (100 × 4.6 mm; 2.6 μm/100 Å) using linear gradients of 10–60% B in A (A = 0.1% FA in water; B = 0.1% FA in acetonitrile) over 20 min and a flow rate of 0.6 mL min^−1^. Further purification of the peptides was achieved by preparative HPLC on RP18 Phenomenex column (Jupiter Proteo, 250 × 15 mm, 4 μm/90 Å) using linear gradients of 10–60% B in A (A = 0.1% TFA in water; B = 0.1% TFA in acetonitrile) over 45 min and a flow rate of 6 mL min^−1^. All peptides were obtained with purities >95%.

### 3.3. FDR Coupling

The coupling reaction was performed in ammonium acetate buffer 0.2 mol·L^−1^ at pH 4.6. 1 eq. of the peptide was dissolved in 100 μL buffer (concentration of peptide 2 µM) and 2 eq. of the 5-FDR- water solution were added. The reaction was left shaking for 20 min at 99 °C [38] (see also, Table 3).

### 3.4. CD Spectroscopy

CD spectra were recorded from 260 nm to 190 nm at 20 °C using a Jasco J-715 spectropolarimeter purged with N_2_ gas. Peptide samples were dissolved in 10 mmol·L^−1^ sodium phosphate buffer (pH 7) containing 0 or 50% (*v*/*v*) TFE. For measuring CD spectra the peptides were dissolved to a final concentration of 20 μmol·L^−1^. Each measurement was repeated four times using a sample cell with a path length of 0.1 cm. Instrument parameters were: response time 2 s, scan speed 50 nm/min, sensitivity 100 mdeg, step resolution 0.5 nm and bandwidth 1.0 nm. All CD spectra were corrected by subtraction of the CD spectrum of the solvent to eliminate the interference from cell, solvent, and optical equipment. 

### 3.5. Cell Culture

For all cellular assays the cell lines used were Capan-1 and HT-29. All cell experiments were carried out under a laminar flow hood Herasafe. The temperature (37 °C) of all used chemicals was adjusted by Julabo SW22 heating bath. Cell culture was carried out at 5% CO_2_ at 37 °C, using 100 × 20 mm Petri dishes.

The medium used for the Capan-1 cells was Dulbecco’s modified Eagle’s medium (90%) with fetal bovine serum (FBS, 10%) and for the HT-29 cells McCoy’s 5A medium (90%) with fetal bovine serum (FBS, 10%).

The cells were cultured up to a density of about 70% (Capan-1) or 80% (HT-29). Subsequently, the medium was removed and the cells washed twice with Dulbecco’s phosphate-buffered saline. For detaching the cells 1 mL Trypsin-EDTA solution was evenly distributed on the cells. After 3 min incubation at 37 °C, the cells were resuspended in 9 mL of medium. This concentrated cell suspension was used for all other tests. In order to determine the cell concentration of the solution, a hemocytometer was used: 10 μL of cell solution was added and the number of cells was manually counted. This cell number was multiplied by 10^4^ to obtain the concentration per milliliter.

### 3.6. Cytotoxicity Assay

In order to test the influence of the peptides on cell viability, a resazurin-based cytotoxicity assay was performed. 96-well plates were used. First, a cell suspension with a defined concentration (for HT-29 60,000 cells per well; for Capan-1 80,000 cells per well) was placed in the wells and filled with medium (with fetal calf serum, +FCS) reaching a final volume of 200 μL. After 24 h or after reaching a confluence of 60–80% the culture medium was replaced by 100 μL of culture medium (+FCS) with a defined peptide concentration. Cells were incubated for 24 h with the peptide solution. After removing the peptide solution, the cells were washed with Dulbecco’s phosphate-buffered saline. Subsequently, the cells were incubated with 10 μL resazurin in 90 μL medium (without fetal calf serum, −FCS) for 1 h. As negative and positive controls, untreated and treated cells (10 min with 70% EtOH in H_2_O) were used. The fluorometrical measurement was performed on a Tecan infinite M200 (Männedorf, Switzerland) at 596 nm with excitation at 550 nm. The toxicity of the peptides was determined for the concentrations 10, 50, and 100 μmol·L^−1^.

### 3.7. Synthesis of the Precursor ***5***

*1-O-Methyl-2,3-O-isopropylidene-5-O-[5-(N,N-dimethylamino)naphthalene-1-sulfonyl]-d-ribofuranoside*: Dansyl chloride (2.9 g, 10.75 mmol) was added to a stirred ice-cold solution of 1-*O*-methyl-2,3-*O*-isopropylidene-d-ribofuranoside (1.7 g, 8.32 mmol) and triethylamine (1.6 g, 15.81 mmol) in CH_2_Cl_2_ (25 mL), the cooling bath was removed and the reaction mixture was stirred for further 16 h. The mixture was concentrated under reduced pressure. Et_2_O and H_2_O (100 mL each) were added to the residue, the organic fraction was separated and washed with H_2_O (3 × 20 mL), 5% NaHCO_3_ (3 × 20 mL) and brine (2 × 20 mL), then dried and concentrated under reduced pressure. The residue was purified by column chromatography [EtOAc/hexane = 1:4, silica gel (0.1% CaO)] and recrystallization from Et_2_O/pentane gave the desired product (2.85 g, 78%) as a yellow solid. Analysis of the compound was in good agreement to the data recently obtained [10].

*1-O-Methyl-2,3-O-isopropylidene-5-O-[5-(N,N,N-trimethylammonium)naphthalene-1-sulfonyl]-d-ribofuranoside triflate*: MeOTf (0.251 mL, 0.376 g, 2.29 mmol) was added to a solution of 1-*O*-methyl-2,3-*O*-isopropylidene-5-*O*-[5-(*N,N*-dimethylamino)naphthalene-1-sulfonyl]-d-ribofuranoside (1 g, 2.29 mmol) in CH_2_Cl_2_ (5 mL) in a dry box under Ar. The reaction flask was then removed from the dry box and the reaction mixture was incubated at ambient temperature for three days. Thereafter, Et_2_O (50 mL) was added and the precipitated oil was separated, recrystallized twice from CH_2_Cl_2_/Et_2_O, sonicated with Et_2_O (3 × 20 mL) and thoroughly dried under reduced pressure to give the desired product (1.05 g, 69%) as a colorless foam. ^1^H-NMR (CDCl_3_, 300 MHz): δ = 1.26 (s, 3H), 1.41 (s, 3H), 3.17 (s, 3H), 3.95–4.15 (m, 2H), 4.08 (s, 9H), 4.30 (dt, *J* = 0.6, 14.1 Hz, 1H), 4.50–4.54 (m, 1H), 4.58–4.62 (m, 1H), 4.90 (s, 1H), 7.83 (t, *J* = 8.3 Hz, 1H), 8.0 (dd, *J* = 8.9, 7.6 Hz, 1H), 8.21 (d, *J* = 8.3 Hz, 1H), 8.47 (d, *J* = 8.9 Hz, 1H), 8.89–8.97 (m, 2H); ^13^C-NMR (CDCl_3_, 75.5 MHz): δ = 24.8, 26.3, 55.1, 58.7, 70.5, 81.2, 83.5, 84.7, 109.5, 112.8, 120.6 (q, *J* = 320.1 Hz), 122.7, 125.2, 127.2, 127.6, 129.4, 131.16, 131.22, 133.9, 142.1. ESI HRMS: calcd for C_22_H_30_NO_7_S^+^: 452.1737; found: 452.1739.

*1-O-Methyl-2,3-O-isopropylidene-5-O-[5-(N,N,N-trimethylammonium)naphthalene-1-sulfonyl]-d-ribofuranoside tosylate*: A solution of 1-*O*-Methyl-2,3-*O*-isopropylidene-5-*O*-[5-(*N,N,N*-trimethylammonium)naphthalene-1-sulfonyl]-d-ribofuranoside triflate (0.22 g, 0.36 mmol) in CH_2_Cl_2_ (20 mL) was extracted with saturated aqueous NaBr solution (5 × 8 mL). The organic fractions were dried over MgSO_4_ and concentrated under reduced pressure. The residue was dissolved in acetone (12 mL) and treated with silver tosylate (1.2 eq.). After vigorous shaking for 5 min, the precipitated silver halide was centrifuged off. The supernatant was concentrated under reduced pressure. Recrystallization from acetone/ether gave the desired product as a white solid (0.05 g, 0,08 mmol, 23%). ^1^H-NMR (300 MHz, CDCl_3_) δ (ppm) = 8.93 (d, *J* = 9.0 Hz, 1H), 8.86 (d, *J* = 8.8 Hz, 1H), 8.40 (d, *J* = 7.2 Hz, 1H), 8.16 (d, *J* = 8.1 Hz, 1H), 7.87–7.84 (dd, *J* = 9.0 Hz, *J* = 7.5 Hz, 1H), 7.71–7.67 (m, 3H), 7.06 (d, *J* = 7.9 Hz, 2H), 4.89 (s, 1H), 4.60 (d, *J* = 6.0 Hz, 1H), 4.52 (d, *J* = 5.9 Hz, 1H), 4.29 (t, *J* = 7.1 Hz, 1H), 4.11 (s, 9H), 4.06–3.99 (m, 2H), 3.17 (s, 3H), 2.26 (s, 3H), 1.41 (s, 3H), 1.26 (s, 3H). ^13^C-NMR (300 MHz, CDCl_3_) δ (ppm) = 143.2 (s, C-4), 142.3 (s, C-27), 139.7 (s, C-13), 133.7 (s, C-24), 131.0, 131.0, 129.6, 128.9 (d, C-7), 128.8 (d, C-25, C-25’), 127.5 (d, C-11), 127.0 (d, C-6), 125.7 (d, C-26, C-26’), 125.2 (s, C-9), 120.8 (d, C-5), 112.8 (s, C-17), 109.4 (C-21), 84.7 (d, C-20), 83.4 (d, C-15), 81.2 (d, C-16), 70.2 (t, C-14), 58.7 (q, C-1, C-2, C-3), 55.0 (q, C-22), 26.2 (q, C-18), 24.8 (q, C.19), 21.1 (q, C-23). ESI HRMS: calcd for C_22_H_30_NO_7_S^+^: 452.1737; found: 452.1739.

### 3.8. Radiochemistry

#### 3.8.1. Preparation of 5-[^18^F]Fluoro-5-Deoxy-d-Ribofuranoside (5-[^18^F]FDR, [^18^F]**7**)

[^18^F]Fluoride was produced via the ^18^O(p,n)^18^F reaction by bombardment of enriched [^18^O]H_2_O with 16.5 MeV protons using MC16 cyclotron (Scanditronix, Uppsala, Sweden) cyclotron. Aqueous [^18^F]Fluoride (200–350 MBq) was trapped on a SepPak Light Waters Accell^TM^ Plus QMA cartridge (Waters GmbH, Eschborn, Germany), washed with methanol (2 mL), and eluted with an onium salt precursor **6** in methanol (500 µL). Methanol was evaporated under argon stream at 60 °C and 500 mbar within 3–5 min. After cooling to room temperature acetonitrile (400 µL) was added and stirred at 120 °C for 20 min. The resulting solution was cooled to room temperature, diluted with 50% acetonitrile and injected into reverse-phase column (Phenomenex Luna C18(2), 250 × 4.6 mm, 5 µm, 1.3 mL/min). The intermediate [^18^F]**6** was eluted with 55% acetonitrile into a second reaction vessel containing 1 mol·L^−1^ HCl (200 µL). The reaction mixture was heated at 110 °C for 12 min. The resulting solution was concentrated under reduced pressure, diluted with ammonium acetate buffer (1–2 mL, 0.2 mol·L^−1^ pH 4) or anilinium buffer (1–2 mL, 0.25 mol·L^−1^, pH 4.6).

#### 3.8.2. Conjugation of 5-[^18^F]FDR to Aminooxy Functionalized Peptides

The isolated 5-[^18^F]FDR (30–60 MBq in 200 µL of the appropriate buffer) was incubated with aminooxy functionalized peptides (1–1.6 µmol in 200 µL of the appropriate buffer) in ammonium acetate buffer (0.2 mol·L^−1^, pH 4,) at 75 °C for 20 min or in anilinium buffer (0.25 mol·L^−1^, pH 4.6) at rt for 10 min. The resulting solution was analyzed by HPLC (Figure 6). ^18^F-labeled compounds were identified by co-injection of the unlabeled reference compounds. [^18^F]FDR-AOA-Clone 27 ([^18^F]**11**, 30 MBq, 15% RCY EOB) was isolated using the same column and chromatographic conditions which were applied for analytic HPLC.

#### 3.8.3. Molar Activity Calculation

The molar activity (GBq/μmol) was calculated by dividing the radioactivity of the purified [^18^F]FDR-AOA-Clone 27 by the amount of the unlabeled tracer determined from the peak area in a UV-HPLC chromatograms (λ = 210 nm). The amount of unlabeled compound was determined from the UV absorbance/concentration calibration curve. The peak area was determined and the amount of carrier was calculated according to the calibration curve.

## 4. Conclusions

In this work, we developed a simple and efficient procedure for the preparation of radiofluorinated claudin-4 binding peptides under ambient conditions using oxime ligation with 5-[^18^F]FDR. We demonstrated that the corresponding peptide conjugates are non-toxic to cells. Consequently, their ^18^F-isotopologues can potentially be used for PET-imaging of claudin-4 overexpressing pancreatic tumors. The clinical application of claudine-4 PET imaging could improve diagnosis of pancreatic cancer at an early stage and, ultimately, reduce mortality from this disease.

## Figures and Tables

**Table 1:** Peptide codes, sequences, and analytical data of the peptide conjugates obtained after 5-FDR coupling. (5-FDR: 5-fluoro-5-deoxy-d-ribose; AOA: aminooxyacetic acid; βA: β-alanine; RT: retention time, for details, see experimental part).

Compound Number	Peptide	Sequence	MW_calc._ [Da]	MW_exp_ [Da]	RT [min]	Purity (%)
**1**	5-FDR-C-CPE-17-KKK	5-FDR = AOA-βA-NSSYSGNYPYSILFQKFKKK	2676.85	2677.17	8.2	99
**2**	5-FDR-M19	5-FDR = AOA-βA-NAPYRGHYPYHILFQKF	2428.29	2428.89	8.0	99
**3**	5-FDR-CC4P-5	5-FDR = AOA-βA-SPWSEPAYTLAP	1595.39	1596.12	9.7	84
**4**	5-FDR-Clone 27	5-FDR = AOA-βA-KTLLPTP	1046.41	1046.45	7.8	88

**Table 2:**
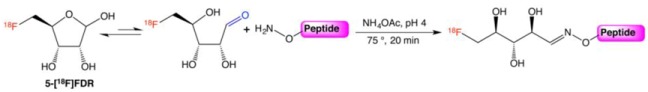
Conjugation of 5-[^18^F]FDR with aminooxy-functionalized peptides ^a^.

Conjugate	Peptide Precursor	Amount Used (µmol)	RCC ^b^ (%)
[^18^F]**8**	AOA-C-CPE-17KKK	1.0	59
[^18^F]**9**	AOA-M19	1.6	46
[^18^F]**10**	AOA-CC4P-5	1.6	60
[^18^F]**11**	AOA-Clone 27	1.0	91

^a^ Conditions: 5-[^18^F]FDR (30–60 MBq), 0.2 mol·L^−1^ NH_4_OAc (pH 4), 75 °C, 20 min; ^b^ RCCs refer to the amount of 5-[^18^F]FDR which was converted into the desired ^18^F-labeled conjugate.

**Table 3:**
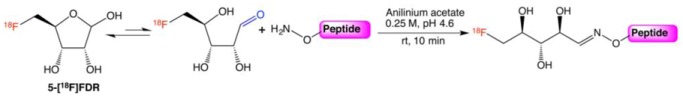
Conjugation of 5-[^18^F]FDR with aminooxy-functionalized peptides in anilinium acetate buffer ^a^.

Conjugate	Peptide Precursor	Amount Used [µmol]	RCC ^b^ [%]
[^18^F]**8**	AOA-C-CPE-17KKK	1.0	76
[^18^F]**9**	AOA-M19	1.6	79
[^18^F]**10**	AOA-CC4P-5	1.6	77
[^18^F]**11**	AOA-Clone 27	1.0	93

^a^ Conditions: 5-[^18^F]FDR (30–60 MBq), anilinium acetate buffer (0.25 mol·L^−1^, pH 4.6), rt, 10 min; ^b^ RCCs refer to the amount of 5-[^18^F]FDR which was transformed into the desired ^18^F-labeled conjugate.

## Supplementary Materials

The following are available online at www.mdpi.com/1424-8247/10/4/99/s1, NMR spectra and HPLC chromatographs of precursors and radiolabeled compounds.

## References

[1] Siegel R.L., Miller K.D., Jemal A. (2016). Cancer Statistics. CA Cancer J. Clin..

[2] Brunner T.B., Seufferlein T. (2016). Pancreatic cancer chemoradiotherapy. Best Pract. Res. Clin. Gastroenterol..

[3] Lee E.S., Lee J.M. (2014). Imaging diagnosis of pancreatic cancer: A state-of-the-art review. World J. Gastroenterol..

[4] Kauhanen S.P., Komar G., Seppänen M.P., Dean K.I., Minn H.R., Kajander S.A., Rinta-Kiikka I., Alanen K., Borra R.J., Puolakkainen P.A. (2009). A prospective diagnostic accuracy study of ^18^F-fluorodeoxyglucose positron emission tomography/computed tomography, multidetector row computed tomography, and magnetic resonance imaging in primary diagnosis and staging of pancreatic cancer. Ann. Surg..

[5] Li X.G., Helariutta K., Roivainen A., Jalkanen S., Knuuti J., Airaksinen A.J. (2014). Using 5-deoxy-5-[^18^F]fluororibose to glycosylate peptides for positron emission tomography. Nat. Protoc..

[6] Hermanson G.T. (2008). Bioconjugate Techniques.

[7] Pretze M., Pietzsch D., Mamat C. (2013). Recent trends in bioorthogonal click-radiolabeling reactions using fluorine-18. Molecules.

[8] Li X.G., Autio A., Ahtinen H., Helariutta K., Liljenbäck H., Jalkanen S., roivainen A., Airaksinen A.J. (2013). Translating the concept of peptide labeling with 5-deoxy-5-[^18^F]fluororibose into preclinical practice: ^18^F-labeling of Siglec-9 peptide for PET imaging of inflammation. Chem. Commun..

[9] Li X.G., Haaparanta M., Solin O. (2012). Oxime formation for fluorine-18 labeling of peptides and proteins for positron emission tomography (PET) imaging: A review. J. Fluor. Chem..

[10] Richarz R., Krapf P., Zarrad F., Urusova E.A., Neumaier B., Zlatopolskiy B.D. (2014). Neither azeotropic drying, nor base nor other additives: A minimalist approach to ^18^F-labeling. Org. Biomol. Chem..

[11] Li X.G., Dall’Angelo S., Schweiger L.F., Zanda M., O’Hagan D. (2012). [^18^F]-5-Fluoro-5-deoxyribose, an efficient peptide bioconjugation ligand for positron emission tomography (PET) imaging. Chem. Commun..

[12] Krause G., Winkler L., Mueller S.L., Haseloff R.F., Piontek J., Blasig I.E. (2008). Structure and function of claudins. Biochim. Biophys. Acta.

[13] Morita K., Furuse M., Fujimoto K., Tsukita S. (1999). Claudin multigene family encoding four-transmembrane domain protein components of tight junction strands. Proc. Natl. Acad. Sci. USA.

[14] Takahashi A., Saito Y., Kondoh M., Matsushita K., Krug S.M., Suzuki H., Tirofumi H., Li X., Aoyama H., Matsuhisa K. (2012). Creation and biochemical analysis of a broad-specific claudin binder. Biomaterials.

[15] Furuse M., Tsukita S. (2006). Claudins in occluding junctions of humans and flies. Trends Cell Biol..

[16] Morin P.J. (2005). Claudin proteins in human cancer: Promising new targets for diagnosis and therapy. Cancer Res..

[17] Oliveira S.S., Morgado-Diaz J.A. (2007). Claudins: Multifunctional players in epithelial tight junctions and their role in cancer. Cell. Mol. Life Sci..

[18] Michl P., Barth C., Buchholz M., Lerch M.M., Rolke M., Holzmann K.H., Menke A., Fensterer H., Giehl K., Lohr M. (2003). Claudin-4 expression decreases invasiveness and metastatic potential of pancreatic cancer. Cancer Res..

[19] Kominsky S.L., Argani P., Korz D., Evron E., Raman V., Garrett E., Rein A., Sauter G., Kallioniemi O.-P., Sukumar S. (2003). Loss of the tight junction protein claudin-7 correlates with histological grade in both ductal carcinoma in situ and invasive ductal carcinoma of the breast. Oncogene.

[20] Kominsky S.L., Vali M., Korz D., Gabig T.G., Weitzman S.A., Argani P., Sukumar S. (2004). Clostridium perfringens enterotoxin elicits rapid and specific cytolysis of breast carcinoma cells mediated through tight junction proteins claudin 3 and 4. Am. J. Pathol..

[21] Long H., Crean C.D., Lee W.-H., Cummings O.W., Gabig T.G. (2001). Expression of Clostridium perfringens enterotoxin receptors claudin-3 and claudin-4 in prostate cancer epithelium. Cancer Res..

[22] Nichols L.S., Ashfaq R., Iacobuzio-Donahue C.A. (2004). Claudin 4 protein expression in primary and metastatic pancreatic cancer—Support for use as a therapeutic target. Am. J. Clin. Pathol..

[23] Soini Y., Tommola S., Helin H., Martikainen P. (2006). Claudins 1, 3, 4 and 5 in gastric carcinoma, loss of claudin expression associates with the diffuse subtype. Virchows Arch..

[24] McClane B.A. (1994). Clostridium-Perfringens Enterotoxin Acts by Producing Small-Molecule Permeability Alterations in Plasma-Membranes. Toxicology.

[25] Sonoda N., Furuse M., Sasaki H., Yonemura S., Katahira J., Horiguchi Y., Tsukita S. (1999). Clostridium perfringens enterotoxin fragment removes specific claudins from tight junction strands: Evidence for direct involvement of claudins in tight junction barrier. J. Cell Biol..

[26] Fujita K., Katahira J., Horiguchi Y., Sonoda N., Furuse M., Tsukita S. (2000). Clostridium perfringens enterotoxin binds to the second extracellular loop of claudin-3, a tight junction integral membrane protein. FEBS Lett..

[27] Ling J., Liao H., Ling J., Clark R., Wong M.S., Lo D.D. (2008). Structural Constraints for the Binding of Short Peptides to Claudin-4 Revealed by Surface Plasmon Resonance. J. Biol. Chem..

[28] Kelly K.A., Bardeesy N., Anbazhagan R., Gurumurthy S., Berger J., Alencar H., DePinho R.A., Mahmood U., Weissleder R. (2008). Targeted nanoparticles for imaging incipient pancreatic ductal adenocarcinoma. PLoS Med..

[29] Torres J.B., Knight J.C., Mosley M.J., Kersemans V., Koustoulidou S., Allen D., Kinchesh P., Smart S., Cornelissen B. (2017). Imaging of claudin-4 in pancreatic ductal adenocarcinoma using a radiolabelled anti-claudin-4 monoclonal antibody. Mol. Imaging Biol..

[30] Liu C., Li X., Ma X., Zhu H., Loft M., Liu H., Cheng Z. (2016). Development of PET probes for molecular imaging of tight junction membrane proteins, claudin-4, in ovarian cancer. J. Nucl. Med..

[31] Neesse A., Hahnenkamp A., Griesmann H., Buchholz M., Hahn S.A., Maghnouj A., Fendrich V., Ring J., Sipos B., Tuveson D.A. (2013). Claudin-4-targeted optical imaging detects pancreatic cancer and its precursor lesions. Gut.

[32] Zischler J., Krapf P., Richarz R., Zlatopolskiy B.D., Neumaier B. (2016). Automated synthesis of 4-[F-18]fluoroanisole, [F-18]DAA1106 and 4-[F-18]FPhe using Cu-mediated radiofluorination under “minimalist” conditions. Appl. Radiat. Isot..

[B33-pharmaceuticals-10-00099] 33.Radiochemical conversion (RCC) refers to the amount of [^18^F]fluoride which is transformed into the desired ^18^F-labeled compound; it was determined by radio-HPLC. Radiochemical yield(RCY) refers to the decay corrected isolated yield of the radiochemically and chemically pure radiolabeled compound.

[34] Dirksen A., Hackeng T.M., Dawson P.E. (2006). Nucleophilic catalysis of oxime ligation. Angew. Chem. Int. Ed. Engl..

[35] Rayo J., Amara N., Krief P., Meijler M.M. (2011). Live Cell Labeling of Native Intracellular Bacterial Receptors Using Aniline-Catalyzed Oxime Ligation. J. Am. Chem. Soc..

[36] Kohler J.J. (2009). Aniline: A Catalyst for Sialic Acid Detection. Chembiochem.

[37] Mezo G., Szabó I., Kertész I., Hegedüs R., Orbán E., Leurs U., Szilvia Bösze S., Halmos G., Manea M. (2011). Efficient synthesis of an (aminooxy) acetylated-somatostatin derivative using (aminooxy) acetic acid as a ‘carbonyl capture’ reagent. J. Pept. Sci..

[38] Dall’Angelo S., Qingzhi Zhang Q., Fleming I.N., Piras M., Schweiger L.F., O’Hagan D., Zanda M. (2013). Efficient bioconjugation of 5-fluoro-5-deoxy-ribose (FDR) to RGD peptides for positron emission tomography (PET) imaging of alpha(v)beta(3) integrin receptor. Org. Biomol. Chem..

