# Supplementary material for: Convenient Preparation of 18F-Labeled Peptide Probes for Potential Claudin-4 PET Imaging
Source: Pharmaceuticals (Basel). 2017 Dec 18;10(4):99. doi: 10.3390/ph10040099 (PMC5748654; doi:10.3390/ph10040099)
Supplement: Supplementary file 1 [file pharmaceuticals-10-00099-s001.pdf]

# Supporting Information

# Convenient preparation of $^{18}$F-labeled peptide probes suitable for PET imaging of claudin-4 expression

Lucia Feni[1†], M. Aymen Omrane[2,3†], Moritz Fischer[1], Boris D. Zlatopolskiy[2,3,4], Bernd Neumaier*[2,3,4], Ines Neundorf*[1]

# $^{1}$H- and APT-NMR Spectra

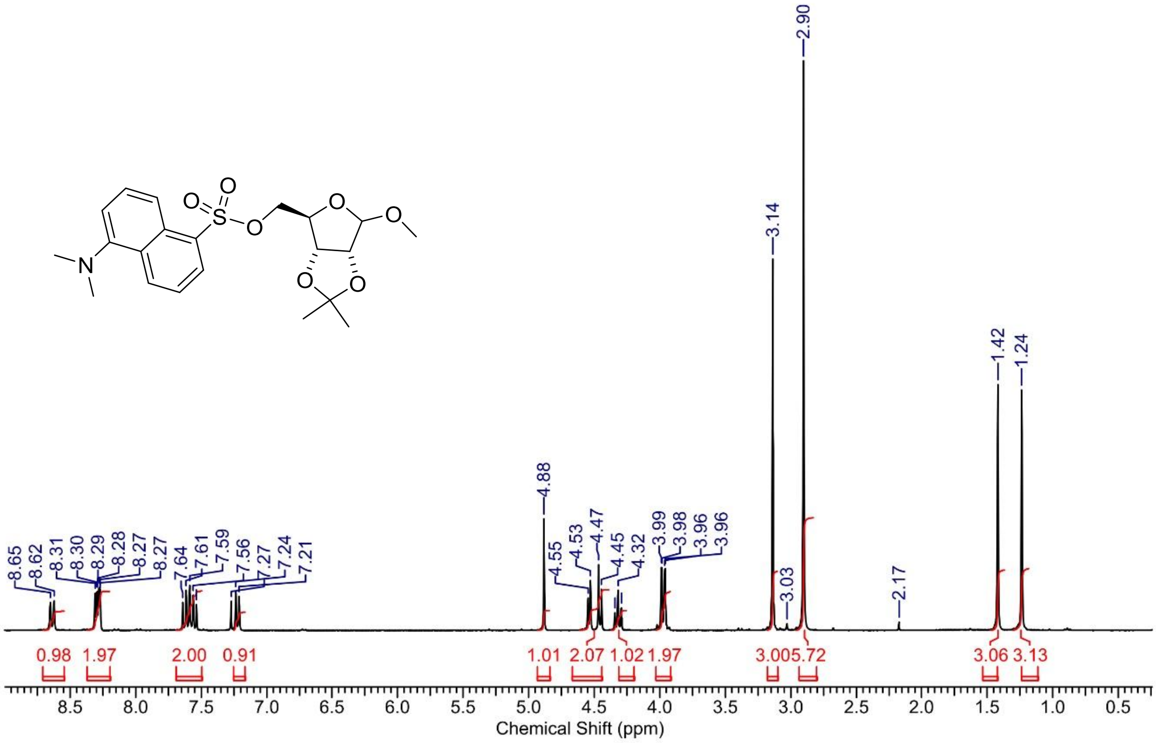


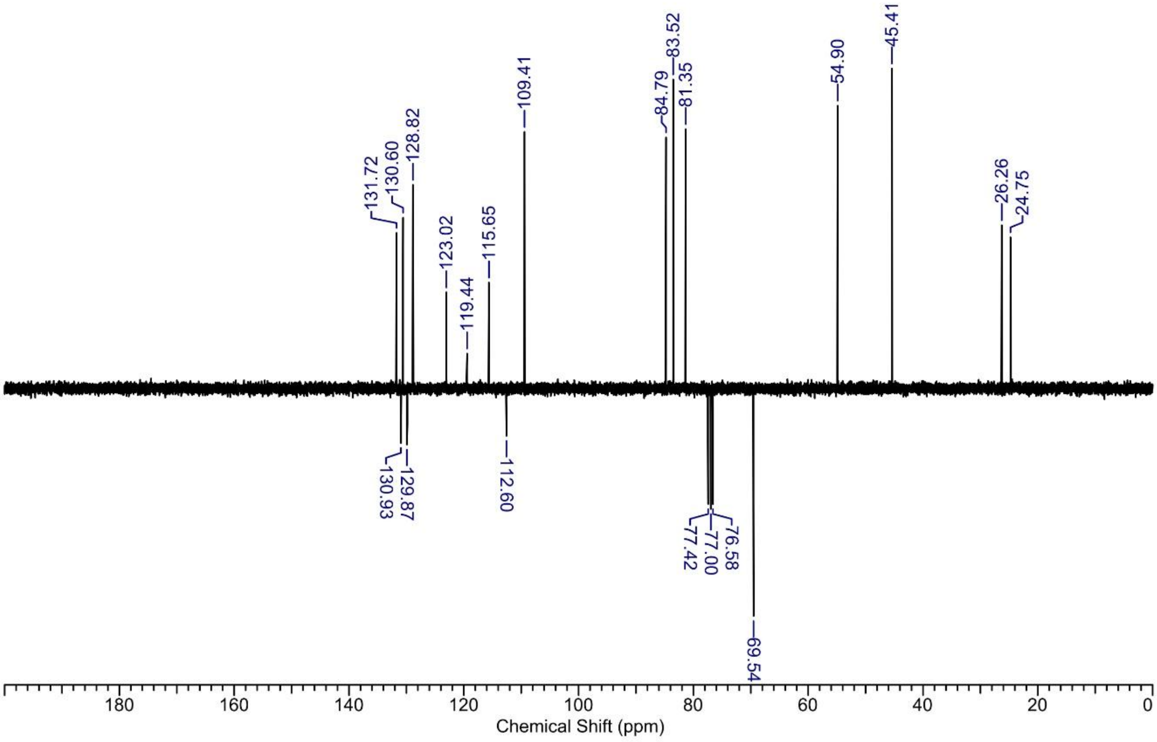

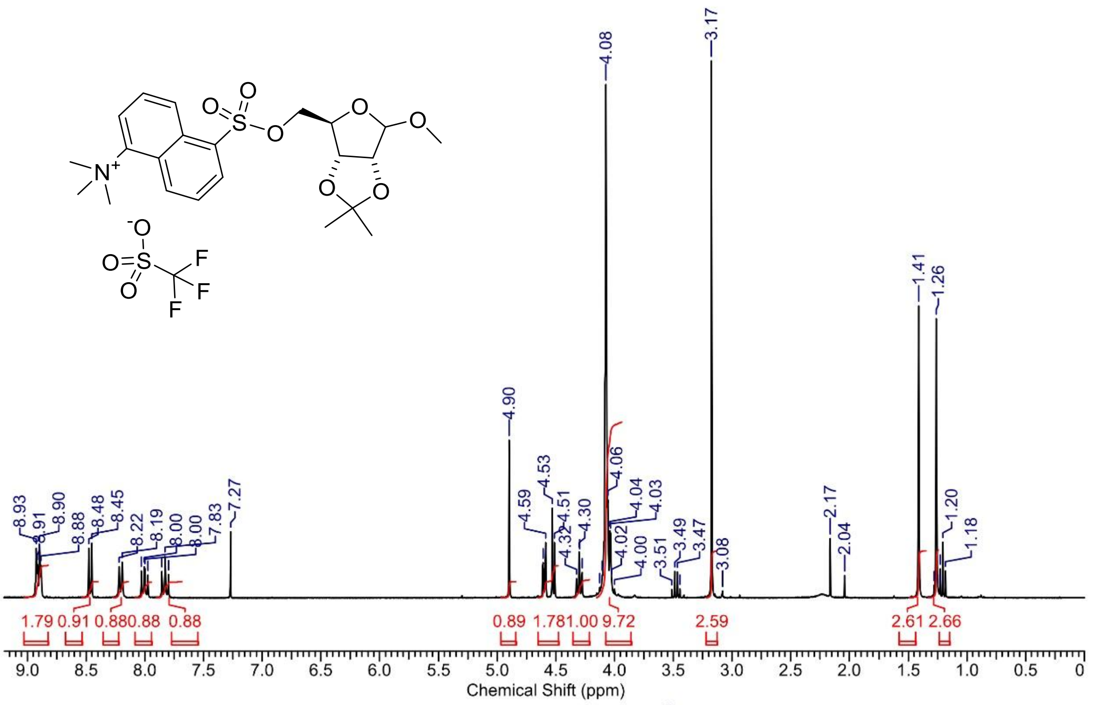
Chemical Shift (ppm)

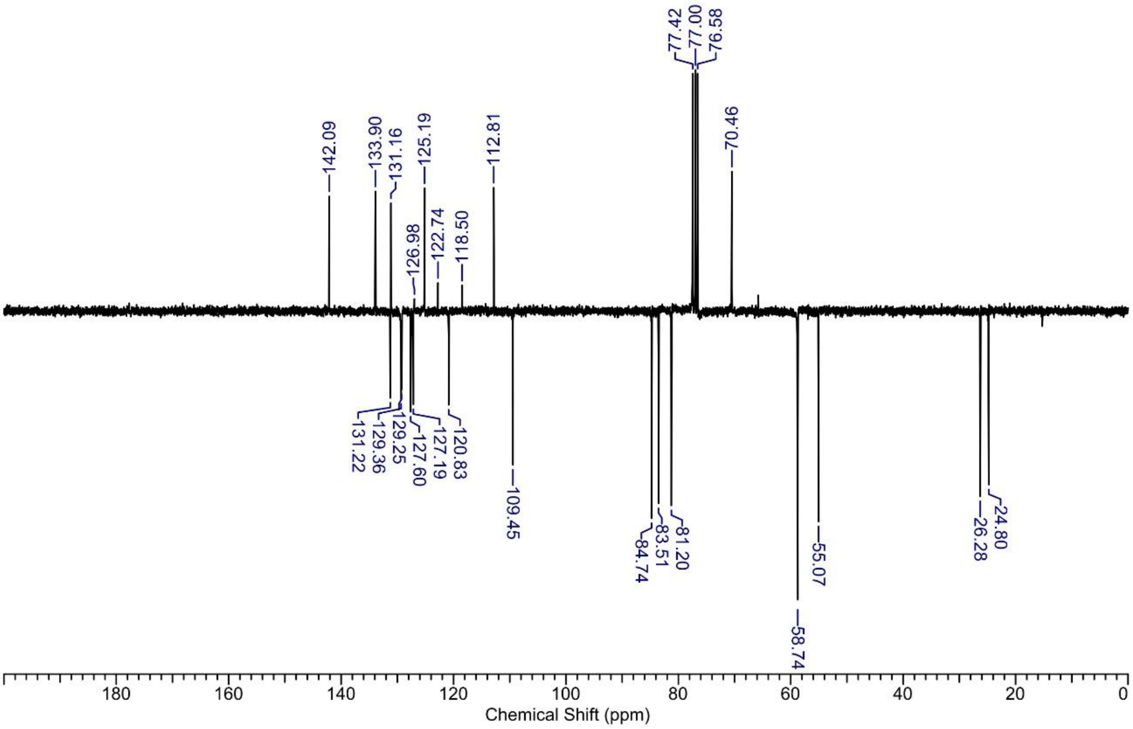
Chemical Shift (ppm)

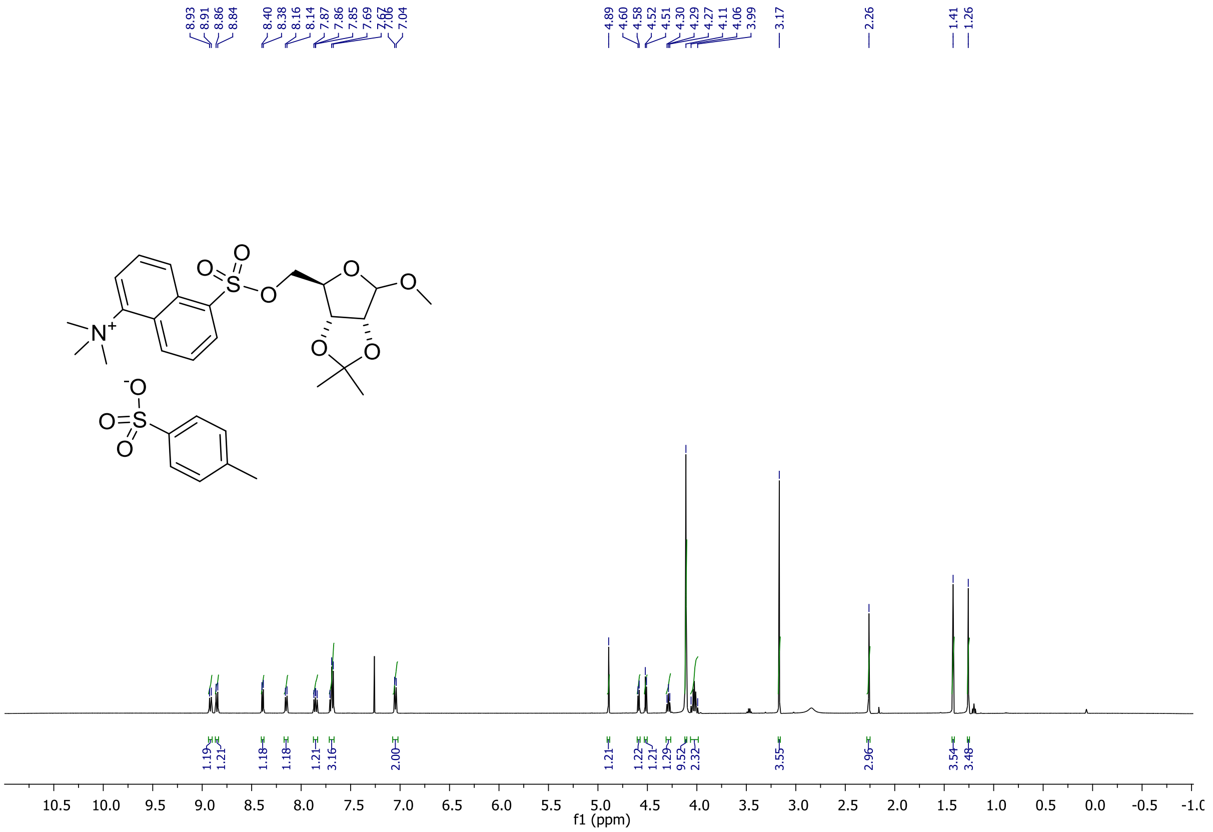
8.93 8.91 8.86 8.84 8.40 8.38 8.16 8.14 7.87 7.86 7.85 7.69 7.67 7.06 7.04
4.89 4.60 4.58 4.52 4.51 4.30 4.29 4.27 4.11 4.06 3.99 3.17 2.26 1.41 1.26
1.19 1.21 1.18 1.18 1.21 3.16 2.00 1.21 1.22 1.21 1.29 9.52 2.32 3.55 2.96 3.54 3.48
f1 (ppm)

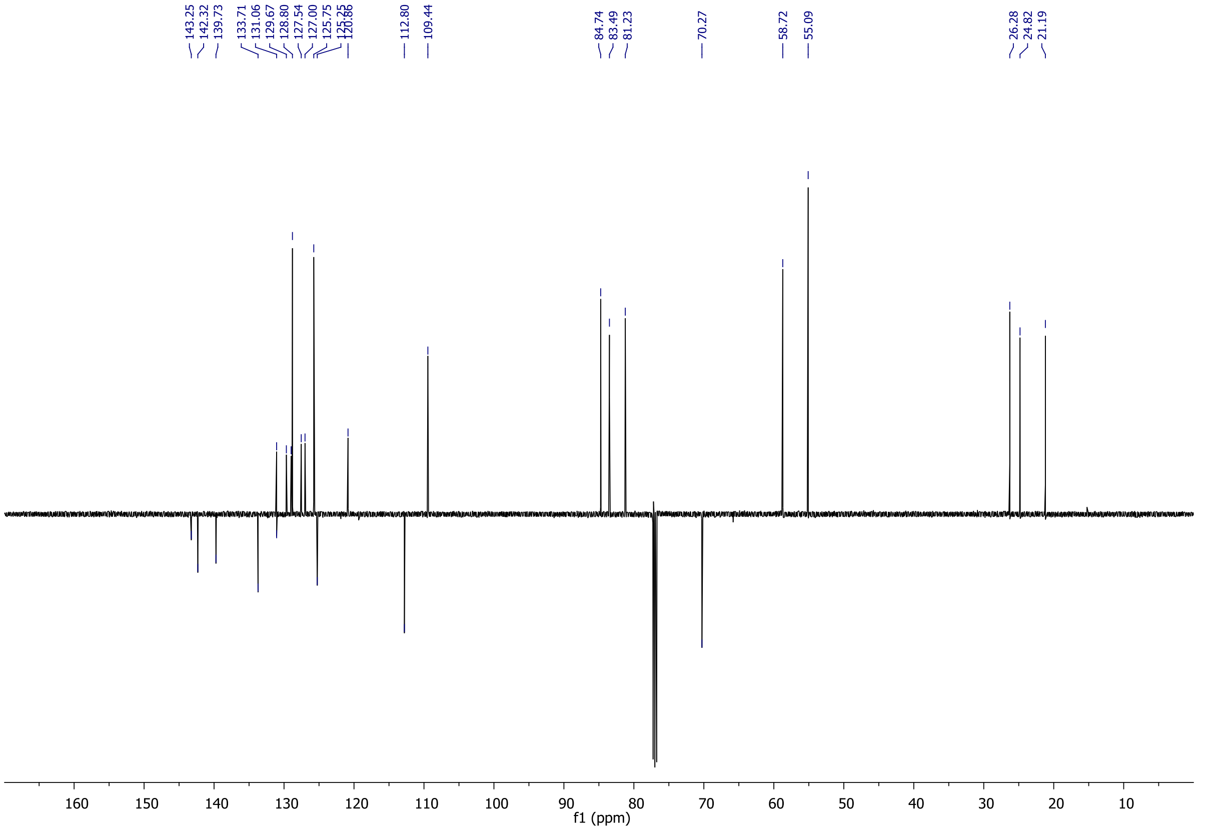
143.25 142.32 139.73 133.71 131.06 129.67 128.80 127.54 127.00 125.75 125.25 120.86 112.80 109.44
84.74 83.49 81.23 70.27 58.72 55.09 26.28 24.82 21.19
f1 (ppm)

## HPLC-Chromatogramms

Protected 5-[$^{18}$F]FDR (crude): column: Phenomenex Luna C18(2), 250 x 4.6 mm, 5µm; eluent: 0-5.5 min 55 → 95% MeCN; 5.5-8.0 min 95% MeCN; flow rate: 1.3 mL/min; $t_R$=4.5 min.

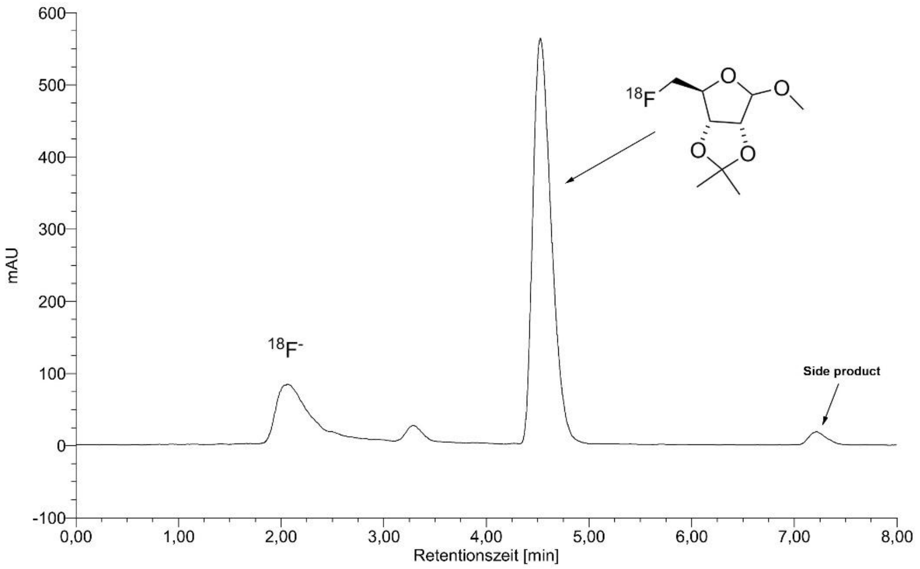


Protected 5-[$^{18}$F]FDR (purified): column: Phenomenex Luna C18(2), 250 x 4.6 mm, 5µm; eluent: 0-5.5 min 55 → 95% MeCN; 5.5-8.0 min 95% MeCN; flow rate: 1.3 mL/min; $t_R$=4.5 min.

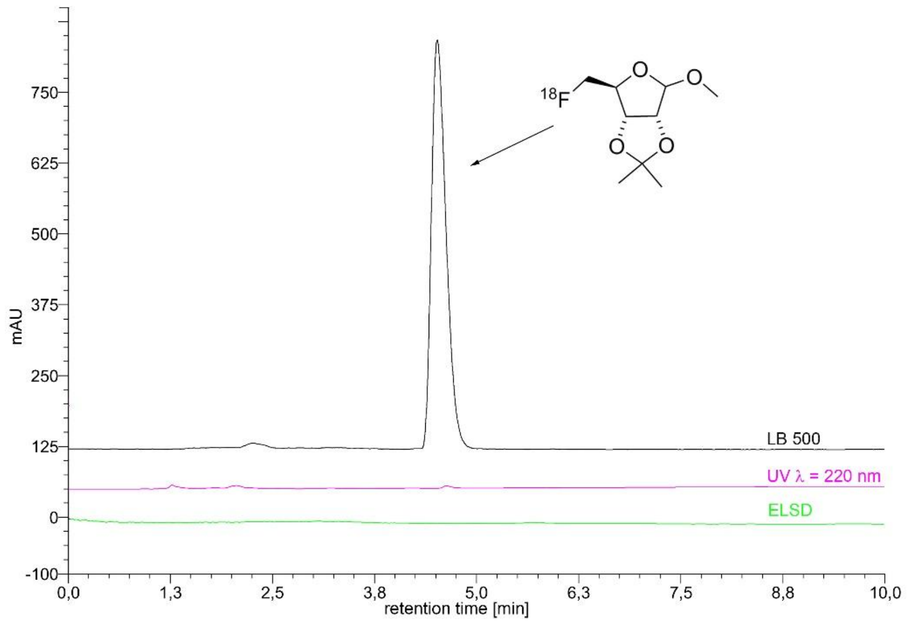


5-[$^{18}$F]FDR: column: Phenomenex Luna C18(2), 250 x 4.6 mm, 5µm; eluent: 0-5.5 min: 55 → 95% MeCN; 5.5-8.0 min: 95% MeCN; flow rate: 1.3 mL/min; $t_R$=2.0 min.

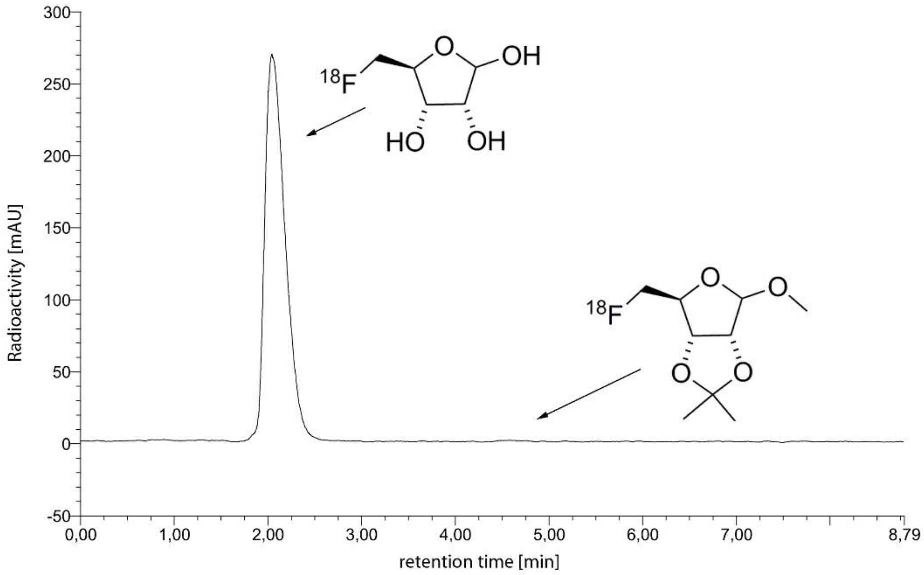


[[18]F]FDR-AOA-C-CPE-17-KKK (crude): column: Phenomenex Kinetex C18 100 x 4.6 mm, 2.6 µm/100 Å; eluent: 0-15 min 10 → 40% MeCN (0.1% TFA); flow rate: 1 mL/min; $t_R$=11.5 min ([[18]F]FDR-AOA-C-CPE-17-KKK), $t_R$=1.4 min ([[18]F]FDR).

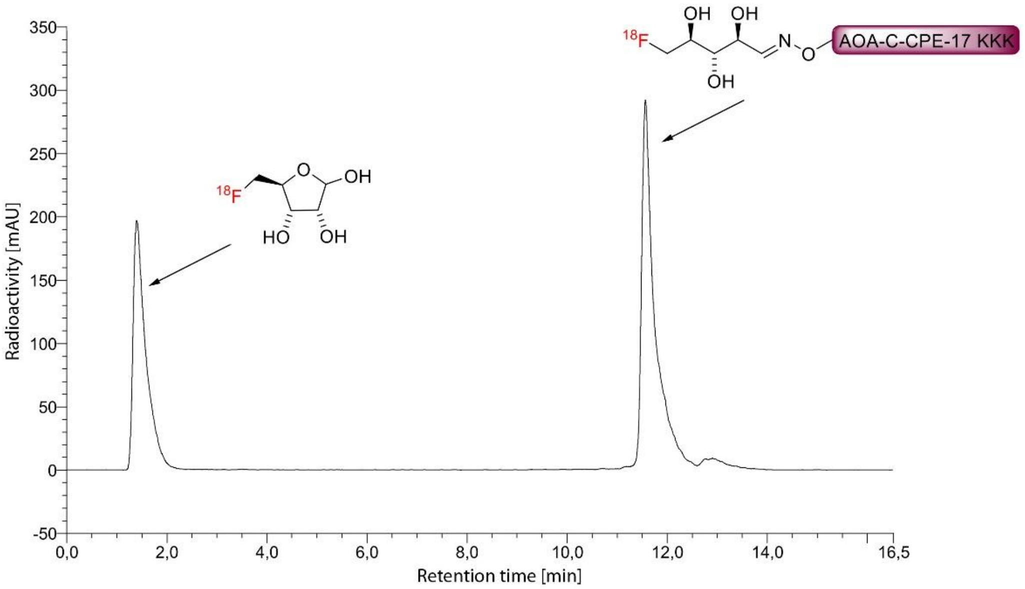


Reaction conditions: $NH_4OAc$ buffer 0.2 M, pH 4 at 75 °C for 20 min.

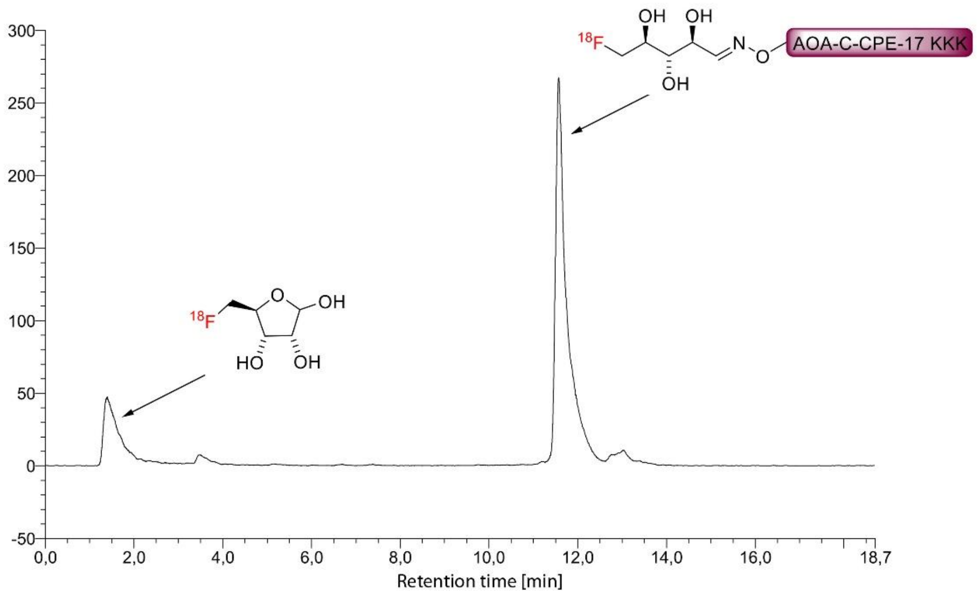


Reaction conditions: anilinium buffer 0.25 M, pH 4.6 at RT for 10 min.

[18F]FDR-AOA-CC4P-5 (crude); column: Phenomenex Kinetex C18 100 x 4.6 mm, 2.6 μm/100 Å; eluent: 0-15 min 10 → 40% MeCN (0.1% TFA); flow rate: 1 mL/min; $t_R$=10.3 min ([18F]FDR-AOA-CC4P-5), $t_R$=1.4 min ([18F]FDR);.

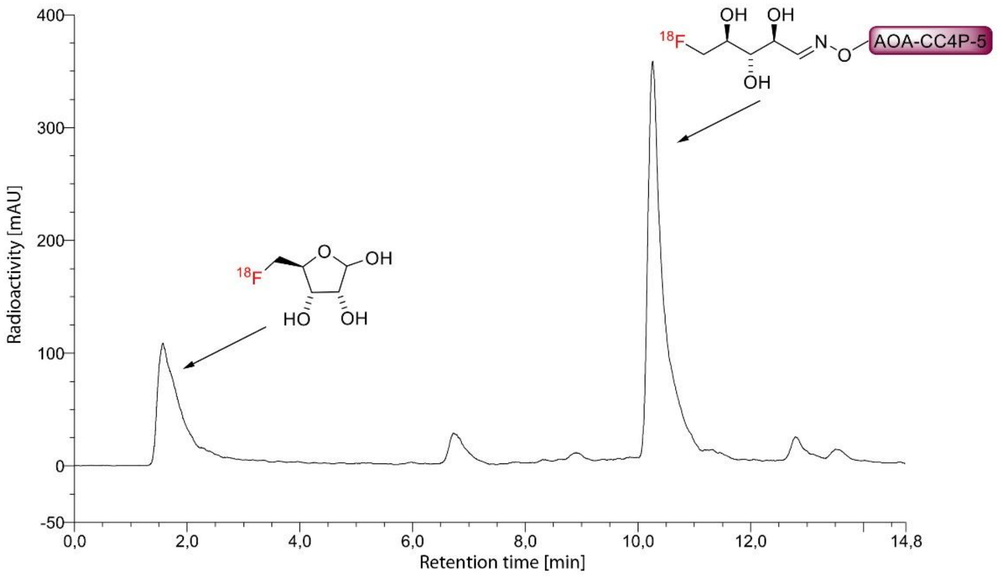


Reaction conditions: $NH_4OAc$ buffer 0.2 M, pH 4 at 75 °C for 20 min.

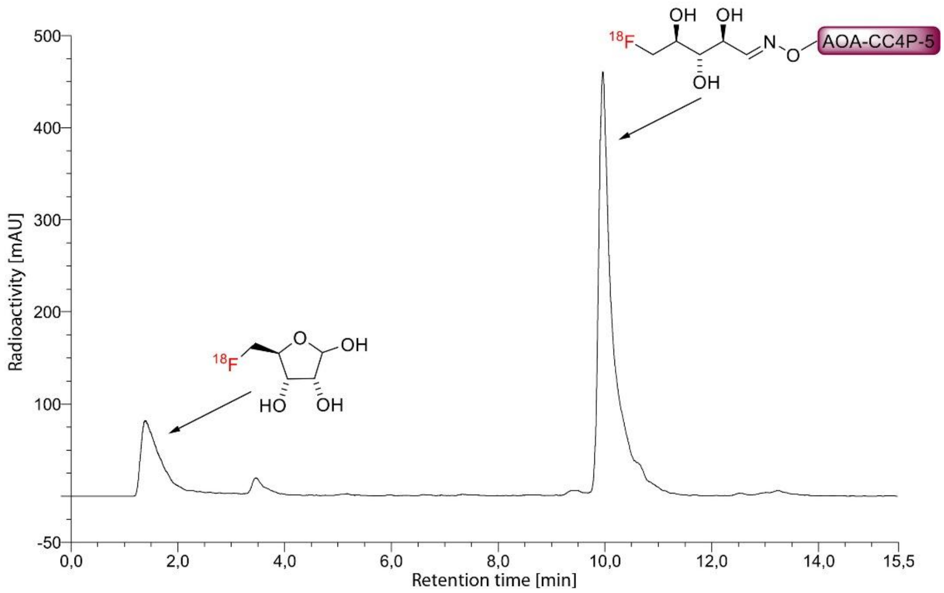


Reaction conditions: anilinium buffer 0.25 M, pH 4.6 at RT for 10 min.

[[18]F]FDR-AOA-Clone 27 (crude); column: Phenomenex Kinetex C18 100 x 4.6 mm, 2.6 µm/100 Å; eluent: 0-15 min 10 → 40% MeCN (0.1% TFA); flow rate: 1 mL/min; $t_R$=7.5 min ([[18]F]FDR-AOA-Clone 27), $t_R$=1.4 min ([[18]F]FDR).

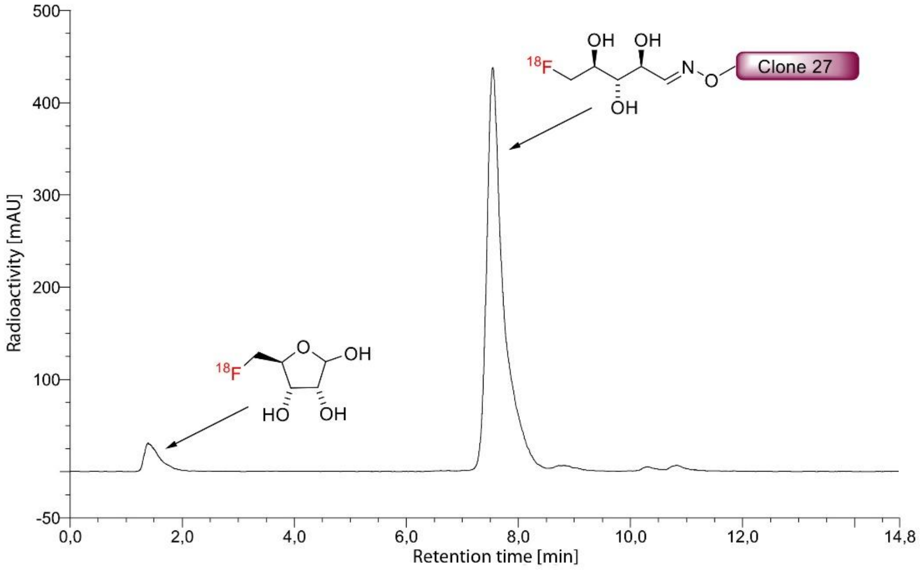


Reaction conditions: $NH_4OAc$ buffer 0.2 M, pH 4 at 75 °C for 20 min.

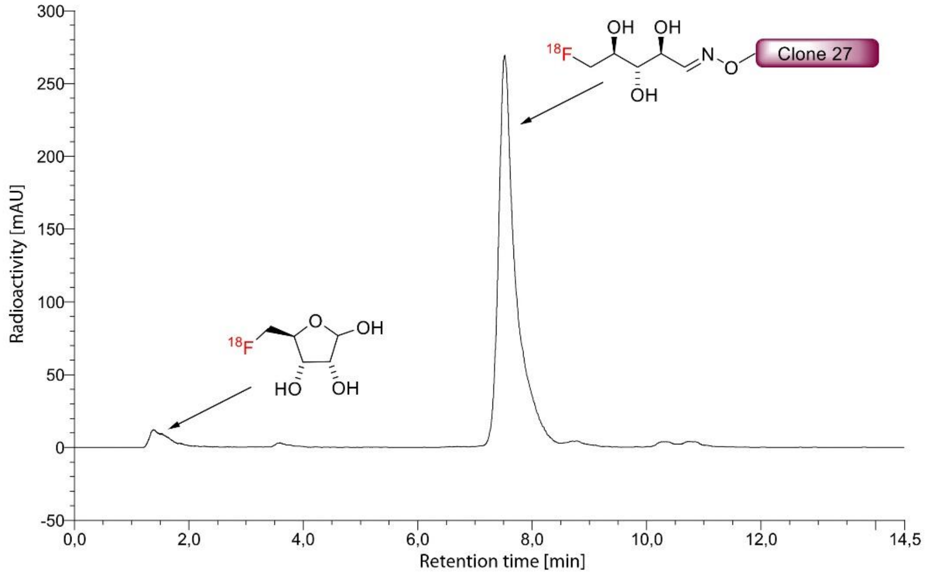


Reaction conditions: anilinium buffer 0.25 M, pH 4.6 at RT for 10 min.

Analytical data of [$^{18}$F]FDR-AOA-M19 (crude); column: Phenomenex Kinetex C18 100 x 4.6 mm, 2.6 µm/100 Å; eluent: 0-15 min 10 → 40% MeCN (0.1% TFA); flow rate: 1 mL/min; $t_R$=11.6 min ([$^{18}$F]FDR-AOA-M19), $t_R$=1.4 min ([$^{18}$F]FDR).

Reaction conditions: anilinium buffer 0.25 M, pH 4.6 at RT for 10 min.

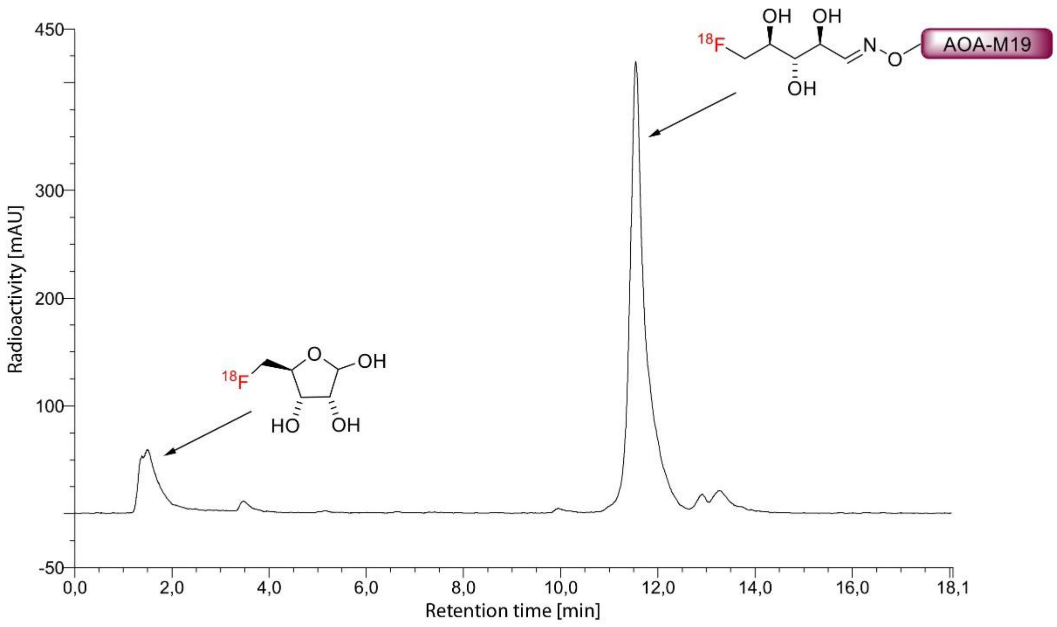

**Molar activity calculation**

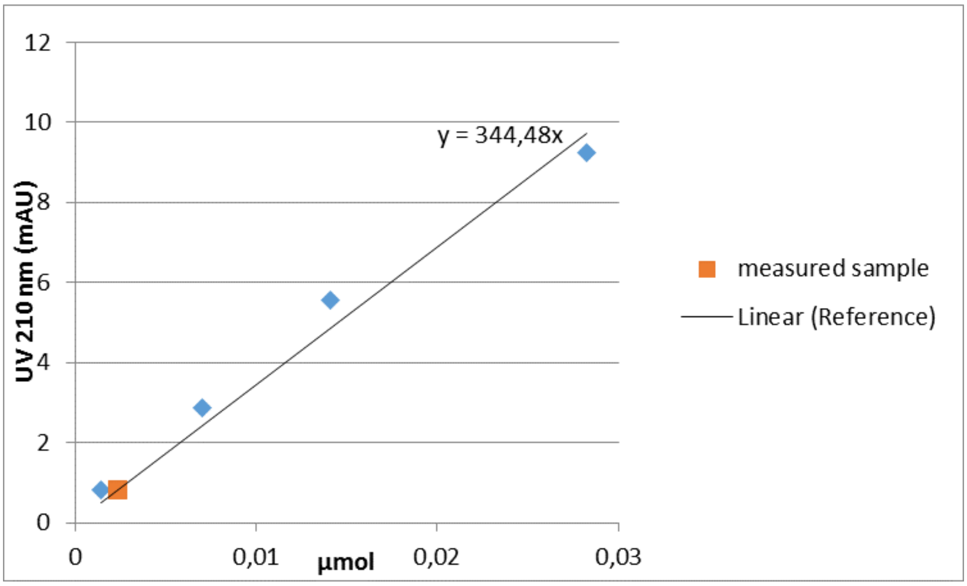


The molar activity (GBq/µmol) was calculated by dividing the radioactivity of the purified [$^{18}$F]FDR-AOA-Clone 27 by the amount of the unlabeled tracer determined from the peak area in a UV-HPLC chromatograms ($\lambda$=210 nm). The amount of unlabeled compound was determined from the UV absorbance/concentration calibration curve. The peak area was determined and the amount of carrier was calculated according to the calibration curve.

**Table S1.** Peptide codes, sequences and analytical data of the peptide conjugates obtained after AOA coupling. (AOA: aminooxyacetic acid; βA: β-alanine; for details, see experimental part)

| Peptid | Sequence | $MW_{calc.}$ | $MW_{exp.}$ | Retention time (min) | Purity (%) |
|---|---|---|---|---|---|
| **AOA-C-CPE-17-KKK** | AOA-βA-NSSYSGNYPYSILFQKFKKK | 2542.9 | 2543.9 | 7.8 | >99 |
| **AOA-M19** | AOA-βA-NAPYRGHYPYHILFQKF | 2294.6 | 2295.4 | 7.7 | >99 |
| **AOA-CC4P-5** | AOA-βA-SPWSEPAYTLAP | 1461.6 | 1461.4 | 9.5 | >90 |
| **AOA-Clone 27** | AOA-βA-KTLLPTP | 912.1 | 912.1 | 7.0 | >90 |
